# ReaxFF-Guided Optimization of VIRIP-Based HIV-1 Entry Inhibitors

**DOI:** 10.1021/acs.jpcb.5c00440

**Published:** 2025-04-03

**Authors:** Fabian Zech, Christoph Jung, Armando Alexei Rodríguez Alfonso, Janet Köhler, Ludger Ständker, Gilbert Weidinger, Timo Jacob, Frank Kirchhoff

**Affiliations:** † Institute of Molecular Virology, Ulm University Medical Center, 89081 Ulm, Germany; ‡ Institute of Electrochemistry, Ulm University, 89081 Ulm, Germany; § Helmholtz-Institute Ulm (HIU) Electrochemical Energy Storage, 89081 Ulm, Germany; ∥ Karlsruhe Institute of Technology (KIT), 76021 Karlsruhe, Germany; ⊥ Core Facility Functional Peptidomics, Ulm University Medical Center, 89081 Ulm, Germany; # Core Unit Mass Spectrometry and Proteomics, Ulm University Medical Center, 89081 Ulm, Germany; ∇ Institute of Biochemistry and Molecular Biology, Ulm University, 89081 Ulm, Germany

## Abstract

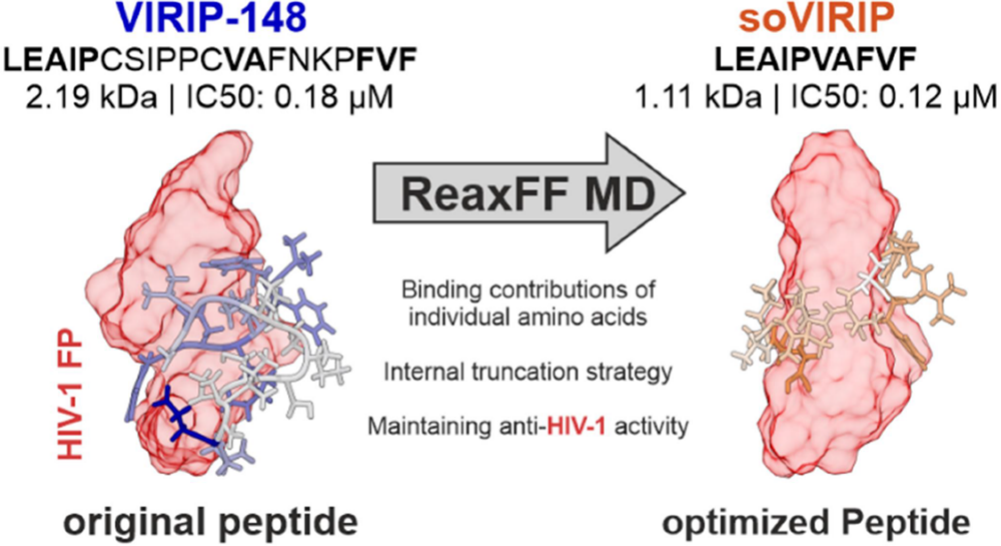

Peptides hold great promise for safe and effective treatment of viral infections. However, their use is often constrained by limited efficacy and high production costs, especially for long or complex peptide chains. Here, we used ReaxFF molecular dynamics (MD) simulations to optimize the size and activity of VIRIP (Virus Inhibitory Peptide), a naturally occurring 20-residue fragment of α1-antitrypsin that binds the HIV-1 GP41 fusion peptide (FP), thereby blocking viral fusion and entry into host cells. Specifically, we used the NMR structure of the complex between an optimized VIRIP derivative (VIR-165) and the HIV-1 gp41 FP for ReaxFF-guided in silico analysis, evaluating the contribution of each amino acid in the interaction of the inhibitor with its viral target. This approach allowed us to reduce the size of the HIV-1 FP inhibitor from 20 to 10 amino acids (2.28–1.11 kDa). HIV-1 infection assays showed that the size-optimized VIRIP derivative (soVIRIP) retains its broad-spectrum anti-HIV-1 capability and is nontoxic in the vertebrate zebrafish model. Compared to the original VIRIP, soVIRIP displayed more than 100-fold higher antiviral activity (IC_50_ of ∼120 nM). Thus, it is more potent than a dimeric 20-residue VIRIP derivative (VIR-576) that was proven safe and effective in a phase I/II clinical trial. Our results show that ReaxFF-based MD simulations represent a suitable approach for the optimization of therapeutic peptides.

## Introduction

The development of peptide drugs, typically composed of short amino acid sequences between 0.5 and 5 kDa, made great progress in recent years and has enormous therapeutic potential.^1,2^ Since the discovery of insulin in 1921, more than 80 peptide drugs have been clinically approved, revolutionizing the treatment of conditions like diabetes.^3^ Early peptide drugs, such as insulin and adrenocorticotropic hormone, were derived from natural sources.^3^ However, advances in protein purification and synthesis during the 20th century facilitated the development and application of synthetic peptides.^2^ Recent technological progress in structural biology and recombinant technologies has further accelerated the development of peptide therapeutics.^4,5^ These drugs span a wide range of therapeutic applications from metabolic and cardiovascular diseases to oncology and antimicrobial treatments.^2,6−8^ More than 170 peptides are currently in clinical development and many more in preclinical stages,^2,3^ highlighting the increasing significance and ongoing innovation in this field.

Peptide drugs are also a promising class of therapeutics against viral pathogens.^9−13^ The entry process of enveloped viruses presents an excellent target for antiviral therapy because it involves several steps that can be interrupted by different classes of inhibitors.^14^ In addition, preventing viral entry reduces the risk of harmful inflammatory responses and cell death.^15,16^ Many viral pathogens enter host cells through similar mechanisms involving receptor binding, fusion peptide exposure, six-helix bundle formation, and membrane penetration.^17,18^ For example, SARS-CoV-2 initially binds to the ACE2 receptor, while HIV-1 first binds to the cluster of differentiation 4 (CD4) receptor and subsequently engages the coreceptors CCR5 or CXCR4. These interactions trigger conformational changes in the respective envelope glycoproteins resulting, in the exposure of the fusion peptide (FP). This hydrophobic peptide then inserts into the host cell membrane, stably anchoring the virion to the target cell.^19,20^ Finally, helical N- and C-terminal heptad-repeat sequences form a six-helix bundle, which pulls the viral and host membranes together to mediate viral entry. The first clinically approved peptidic HIV-1 entry inhibitor, Enfuvirtide (Fuzeon, T20), prevents six-helix bundle formation.^21,22^ More recently, highly potent peptides acting by the same mechanism to inhibit SARS-CoV-2 and other coronaviruses have been reported^23,24^ and are currently evaluated in clinical trials.^25^

The VIRus Inhibitory Peptide (VIRIP) targets the step just before six-helix bundle formation, i.e. insertion of the HIV-1 gp41 FP into the cell membrane.^26^ VIRIP was initially discovered by screening of a human hemofiltrate peptide library and represents a naturally occurring 20 amino acid residue fragment of α1-antitrypsin, the most abundant circulating serine protease inhibitor.^26^ VIRIP specifically binds to the highly conserved FP region of the HIV-1 transmembrane glycoprotein 41 (gp41), thereby blocking its penetration into the host cell membrane and preventing viral entry.^26^ This mechanism came as a surprise because it was previously thought that viral FPs shoot into the membrane like harpoons and that this step is too fast to be targeted.^27^ However, after the identification of the HIV-1 FP as a site of vulnerability, it has been shown that vaccination with the FP induces broadly neutralizing antibodies.^28^ Structure–function analyses allowed to greatly enhance the antiviral activity of VIRIP.^26,29,30^ In addition, VIRIP-based inhibitors proved to be active against all HIV-1 variants and did not show cross-resistance with other antiretroviral drugs. Resistance to improved VIRIP derivatives has a very high genetic barrier and comes at enormous costs for viral replication fitness.^31,32^ Most notably, monotherapy with an optimized analogue (VIR-576) was safe and effective in a phase I/II clinical trial.^33^ However, the necessity of intravenous injection of high doses of VIR-576, together with the availability of various highly active and orally available drugs against HIV-1, made VIRIP-based inhibitors unattractive for broad therapeutic use in humans. To overcome some of these drawbacks and to pave the way for optimization of other peptide drugs with defined targets, we applied a ReaxFF-guided strategy to further optimize the size and activity of VIRIP.

## Results and Discussion

**Figure 1:**
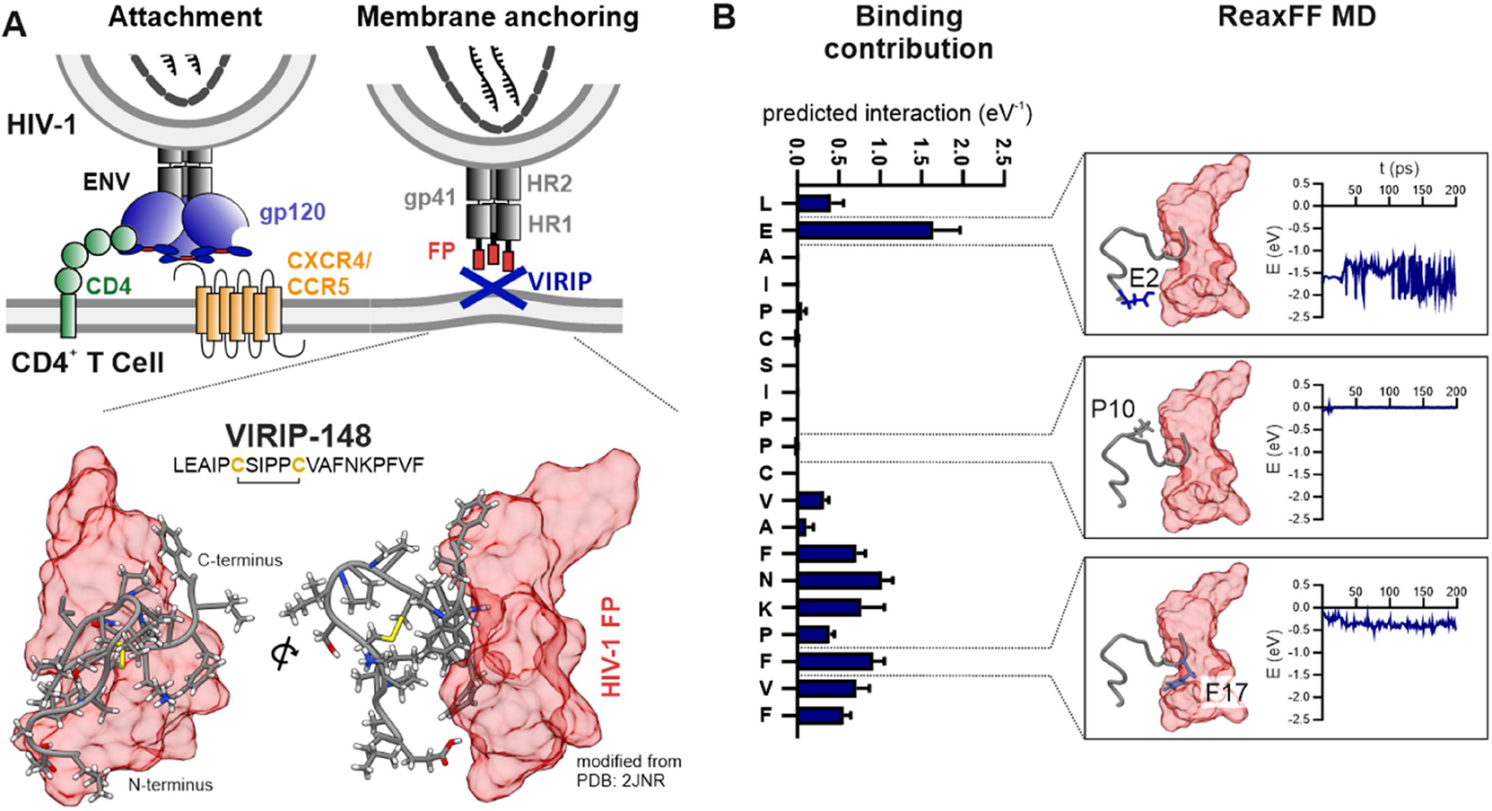
C- and N-terminal interactions mediate binding of VIR-148 to the HIV-1 gp41 FP. (A) Schematic presentation of HIV-1 attachment and membrane anchoring (upper panel). HIV-1 binds to CD4 and a coreceptor (CXCR4 or CCR5) on CD4+ T cells. The viral gp41 FP (red) is blocked by VIRIP. The lower panel shows VIR-148 (modified from PDB 2JNR(26)) bound to the HIV-1 gp41 FP. (B) Predicted contribution of each amino acid in VIR-148 to the interaction energy with gp41 FP (left panel). ReaxFF MD simulations for selected alanine mutations: E2A, P10A, and F17A (right panel).

VIRIP binds to the HIV-1 gp41 FP, thereby preventing the anchoring of the viral particle to the cellular membrane and consequently its entry into the target cells (Figure 1A, upper panel). Based on the NMR structure of VIR-165, an optimized VIRIP derivative, in complex with the HIV-1 gp41 FP (PDB 2JNR),^26^ we utilized the rotamer function of UCSF Chimera to predict the conformation of VIR-148 (Figure 1A, lower panel). The latter differs from VIR-165 by a single amino acid change (F12V) and was selected because it has an IC_50_ of 180 nM, making it the most active VIRIP derivative identified in comprehensive structure–activity-relationship (SAR) studies.^26^ ReaxFF-based molecular modeling revealed that binding of the HIV-1 FP is mainly mediated by amino acids near the N and C termini of VIR-148 (Figure 1B). This mode of binding is supported by alanine exchange SAR studies of the original VIRIP peptide.^26^ It has been previously shown that introduction of cysteine residues substantially enhances the antiviral activity of VIRIP derivatives.^26^ Notably, the NMR structure reveals that establishment of a cysteine bridge forces VIR-148 to form a loop structure that likely stabilizes the conformation allowing effective binding of the HIV-1 gp41 FP (Figure 1A, lower panel).

**Figure 2:**
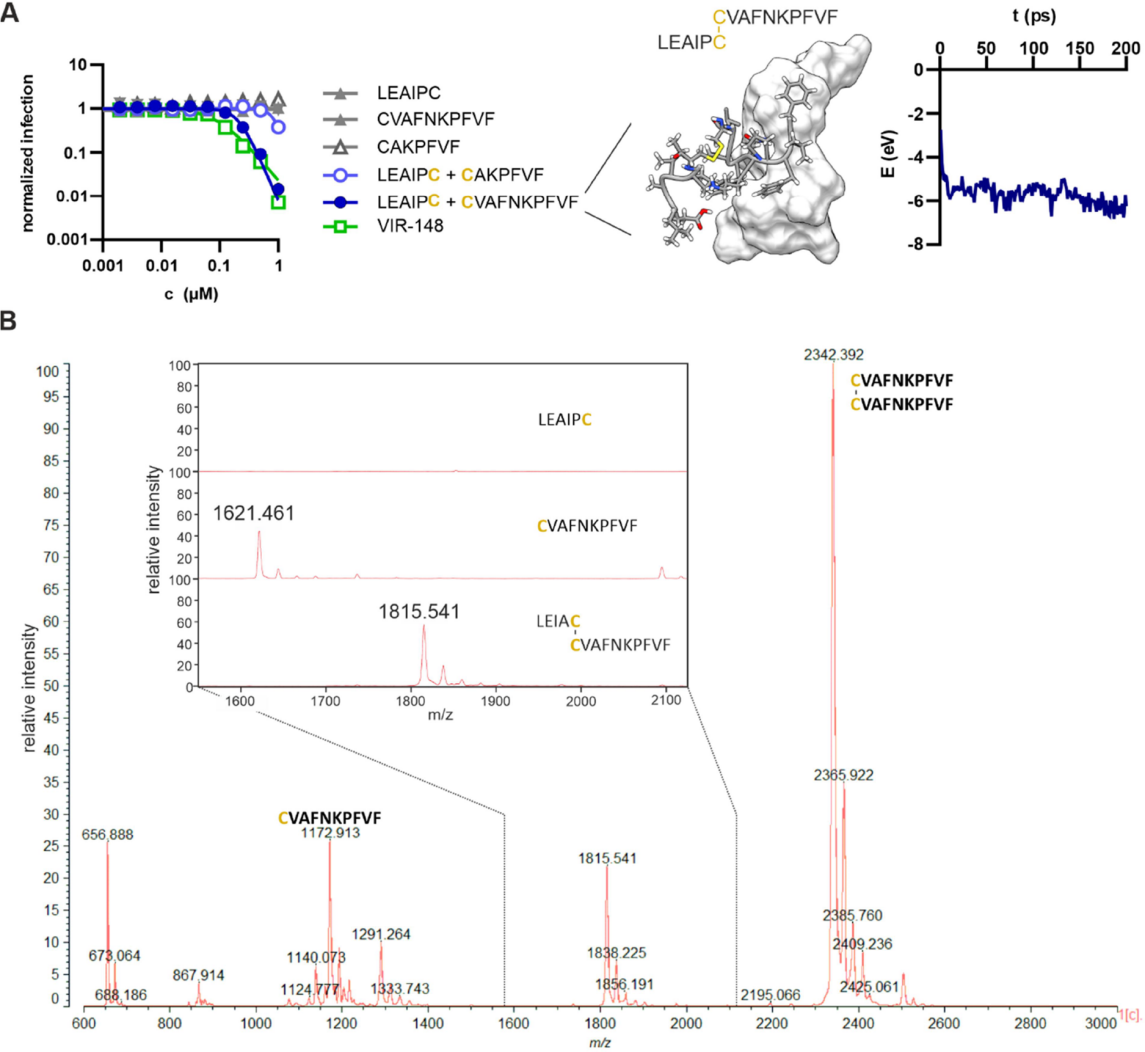
Short N- and C-terminal fragments linked by a cysteine-bridge show efficient anti-HIV-1 activity. (A) TZM-bl reporter cells were incubated with increasing concentrations of VIR-148 or the indicated individual or mixed N- and C-terminal fragments (left). Cells were subsequently infected with HIV-1 NL4-3, and beta-galactosidase activity was determined 3 days postinfection. ReaxFF-based molecular dynamics simulation of the interaction between the indicated C–C-linked short VIR-148 fragments and the HIV-1 gp41 FP (middle). The structure after 150 ps (left) and ReaxFF MD simulation of the total interaction energy between cysteine-bridge-coupled split VIR-148 and the HIV-1 gp41 FP (right). (B) Mass spectrometry analysis of an equimolar mix of the two cysteine-containing VIR-148 fragments in cell culture medium.

To further define critical residues and to assess whether the loop structure is required for antiviral activity, we generated short peptides of six, seven or ten amino acids corresponding to the C- or N-termini of VIR-148, respectively. To determine their antiviral activity, we pretreated TZM-bl reporter cells with various concentrations of individual or mixed peptides and subsequently exposed them to the CXCR4-tropic HIV-1 NL4–3 strain. TZM-bl reporter cells are highly susceptible to HIV-1 infection and contain the β-galactosidase reporter gene under the control of the HIV-1 long terminal repeat.^34^ Three days later, infection was quantified by β-galactosidase assay. Individually, these short peptides did not inhibit HIV-1 infection (Figure 2A, left). In contrast, mixtures of the split VIR-148 fragments, allowing bridging via the cysteines at their C- and N-termini, inhibited HIV-1 infection almost as efficient as the parental intact VIR-148 peptide. In agreement with these results, ReaxFF-based molecular modeling confirmed the ability of the linked N- and C-terminal VIR-148 fragments to stably bind the HIV-1 gp41 FP (Figure 2A, right). Mass Spectrometry confirmed the formation of the heterologous disulfide-bridged LEAIPC–CVAFNKPFVF VIRIP derivative from the two short peptides (Figure 2B). Altogether, these results showed that the N- and C-terminal regions of VIR-148 are both required and sufficient for antiviral activity.

**Figure 3:**
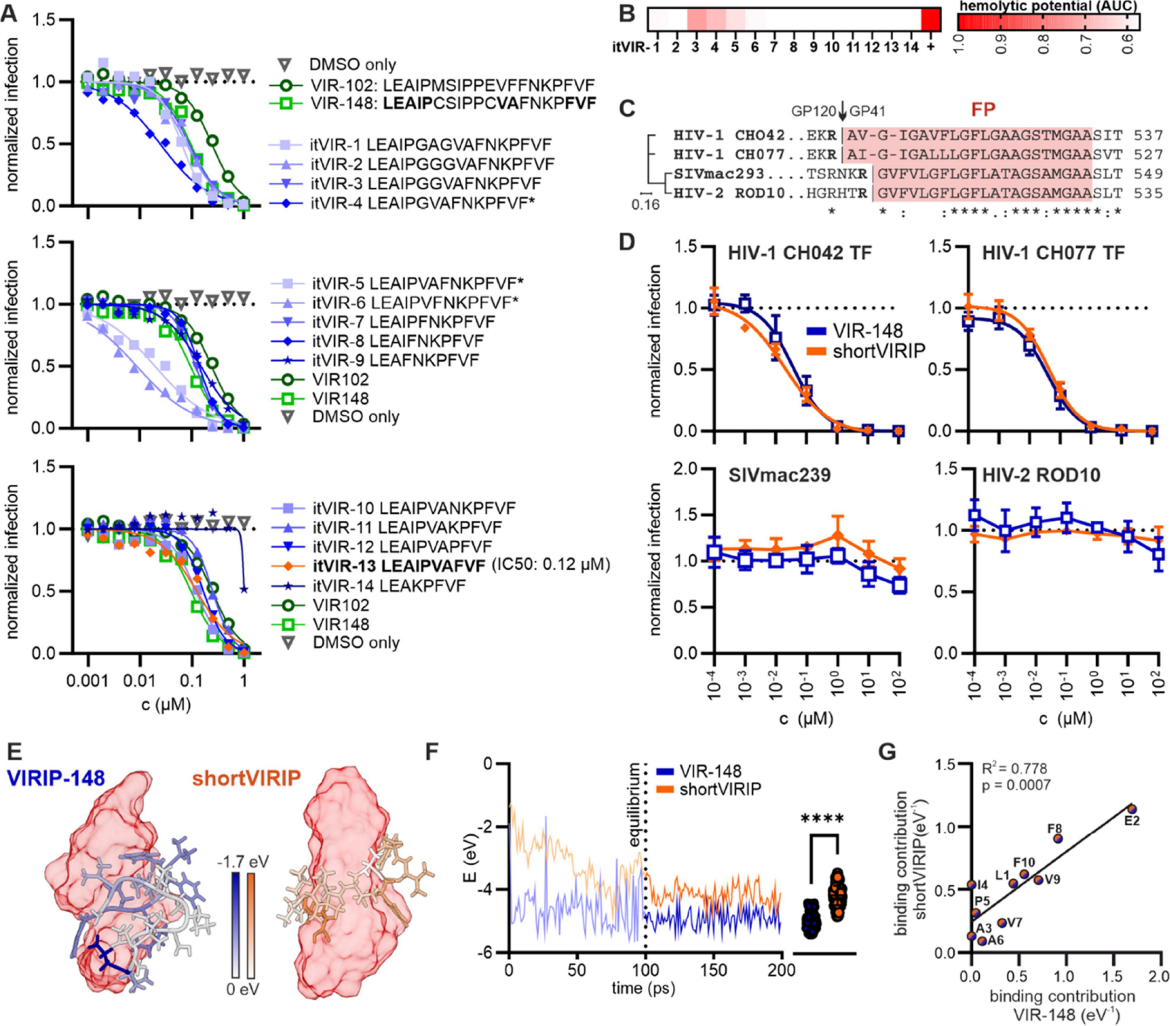
Size optimization of VIR-148. (A) TZM-bl reporter cells were incubated with increasing concentrations of VIR-148, VIR-102, or the indicated internally truncated itVIRIP derivatives. Cells were subsequently infected with HIV-1 NL4-3, and beta-galactosidase activity was determined 3 days after infection. (B) Hemolytic effect of indicated compounds on human erythrocytes. Each tile represents the area under the curve (AUC) for hemolytic activity. Full hemolysis is represented by a Triton-X control. (C) Amino acid alignment of the FP regions (red) of HIV-1 CH042, CH077, SIVmac239 and HIV-2 ROD10. (D) TZM-bl cells were incubated with increasing concentrations of VIR-148 or soVIRIP and subsequently infected with the HIV-1 CH042 or CH077, SIVmac239 or HIV-2 ROD10. (E) Exemplary ReaxFF structure of VIR-148 (blue) and soVIRIP (orange) binding to the HIV-1 gp41 FP (surface, red) after 150 ps of simulation time. The contribution of each amino acid to the binding interaction is indicated by color saturation. (F) Time-resolved ReaxFF-based molecular dynamics simulation of the interaction between soVIRIP and the HIV-1 FP. The trajectories are shown for VIR-148 (blue) and soVIRIP (orange). (G) Correlation between the free energy contribution of each amino acid of soVIRIP with the corresponding amino acid in VIR-148, based on ReaxFF simulations.

After confirming that the loop structure encompassing the internal residues of VIR-148 is not critical for FP binding and antiviral ability, we sequentially deleted the central region of the peptide, thereby gradually reducing its size (Figure 3A). For comparison, we included VIR-102, the most active VIRIP derivative that does not contain a cysteine and is therefore unable to dimerize or stabilize its structure through a disulfide bridge.^26^ The internally truncated VIRIP (itVIR) derivatives itVIR4, 5, and 6 showed high antiviral activity (Figure 3A). However, these derivatives formed visible precipitates and exhibited hemolytic activity (Figure 3B). In contrast, derivatives itVIR-7 through itVIR-13 were well-soluble and did not exhibit hemolytic activity (Figure 3B) while maintaining IC_50_ values close to their VIR-148 precursor (Figure 3A). itVIR-13 (named “size-optimized” soVIRIP hereafter) had only half the size (10 amino acids) and mass (1.11 kDa) compared to the parental VIR-148 peptide but maintained full antiviral activity (Table 1).

**Table 1:**
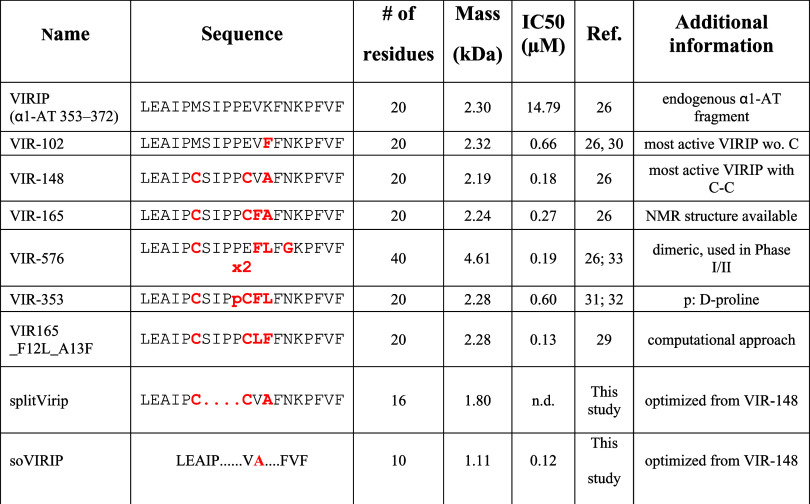
Overview on VIRIP Derivatives^a^

a Amino acid changes compared to the original VIRIP sequence are highlighted in bold, red letters; deletions are indicated by dots.

Further internal truncations, such as in itVIR-14, strongly impaired antiviral activity (Figure 3A, bottom). It has been previously shown that VIRIP and its derivatives inhibit diverse HIV-1 strains but are inactive against HIV-2 and simian immunodeficiency viruses infecting macaques (SIVmac),^26^ which show substantial amino acid diversity in the FP region from that of HIV-1 domain (Figure 3C). In agreement with the previous data, both the original VIR-148, as well as the soVIRIP derivative, efficiently inhibited the clade B CH077 and clade C CH042 transmitted-founder (TF) HIV-1 strains with IC_50_ values of 39 and 37 nM, respectively (Figure 3D). In contrast, both VIRIP derivatives were inactive against HIV-2 ROD10 and SIVmac239, suggesting that the determinants of VIR-148 and soVIRIP interaction with the HIV-1 gp41 FP are conserved. This was confirmed by further ReaxFF-based modeling of VIR-148 and soVIRIP binding to the HIV-1 gp41 FP (Figure 3E). After an equilibration time of 100 ps, the total interaction energy of soVIRIP binding to the HIV-1 FP decreased by 0.25 eV (Figure 3F). Notably, the contribution of individual amino acid residues in VIR-148 and soVIRIP to gp41 FP binding correlates significantly (Figure 3G). Altogether, our in silico analyses and in vitro results suggest that VIR-148 and short VIRIP target the same region in the HIV-1 gp41 FP and bind with similar efficiency.

**Figure 4:**
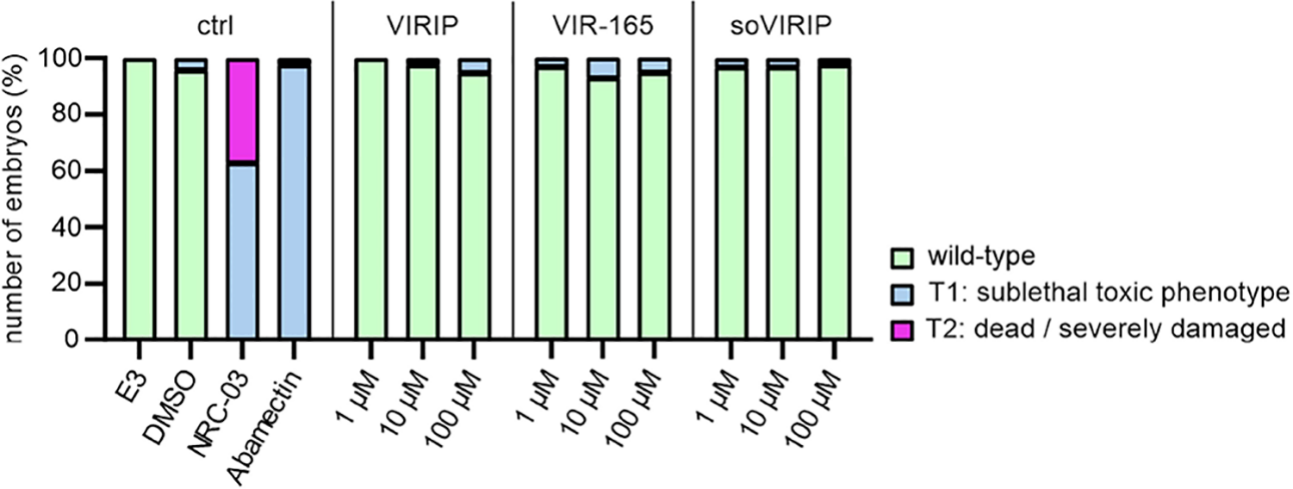
soVIRIP is not toxic to embryonic zebrafish. Twenty-four hours post fertilization, dechorionated zebrafish embryos were exposed to DMSO, NRC-03 (cytotoxic control), abamectin (neurotoxic control), or increasing amounts of the indicated peptide for 24 h. Data shown are derived from 60 embryos per group, sampled in two independent experiments.

To assess potential toxic effects of VIRIP and its optimized derivatives in vivo, we used zebrafish embryos. This model is increasingly used in toxicity studies since most zebrafish organs perform the same functions as their human counterparts. The transparency of the embryos allows for evaluation not only of mortality, but also of sublethal cytotoxicity (necrosis, lysis), developmental toxicity (developmental delay, malformations) or toxicity affecting specific organ systems, in particular cardiotoxicity (heart edema, reduced circulation) and neurotoxicity (reduced touch escape response).^35^ We found that the optimized soVIRIP derivative did not display significant toxic effects in the zebrafish model, whereas NRC-03 (cytotoxic control) and abamectin (neurotoxic control) exhibited the expected toxicities (Figure 4). Our finding that soVIRIP was nontoxic at concentrations about 2,500-fold higher than the IC_50_ against primary HIV-1 strains suggests that it might be well tolerated in vivo.

In the present study, we used ReaxFF-based prediction of amino acids involved in the binding of an optimized VIRIP derivative to the HIV-1 FP to optimize the size of this antiviral peptide fusion. Prediction of the key residues critical for binding by this computational approach allowed us to reduce the size of the antiviral peptide by half without compromising its activity against HIV-1 (Table 1). Such a reduction in peptide size can enhance therapeutic potential by improving its bioavailability, stability, and ease of synthesis, while maintaining the crucial interactions required for antiviral efficacy. Notably, membrane permeability and hence oral availability is size dependent and rapidly increases for peptides with molecular mass of ≤1 kDa,^36^ which is approximated by soVIRIP (1.1 kDa). Our results provide proof-of-concept that ReaxFF-based predictions of target binding are a powerful tool for the optimization of peptides for clinical applications. Overall, this approach may allow to reduce the production costs and immunogenicity of other therapeutic peptides, while retaining full activity.

## Material and Methods

### Cell Culture

HEK293T cells were provided and authenticated by the ATCC. TZM-bl reporter cells were provided and authenticated by the NIH AIDS Reagent Program, Division of AIDS, NIAID. HEK293T and TZM-bl cells were maintained in Dulbecco’s modified Eagle medium (DMEM) supplemented with FCS (10%), l-glutamine (2 mM), streptomycin (100 mg/mL) and penicillin (100 U/mL). Cells were cultured at 37 °C, 90% humidity and 5% CO2.

### Virus Stock Production

Virus stocks were generated by transient transfection of HEK293T cells using the calcium-phosphate precipitation method. One day before transfection, 0.8 × 10^6^ HEK293T cells were seeded in 6-well plates (Sarstedt, Germany). At a confluence of 80% cells were used for transfection. For the calcium-phosphate precipitation method, 5 μg of the proviral HIV-1 constructs NL4-3,^37^ CH055_TF1, CH077_TF1,^38^ as well as HIV-2 ROD10^39^ and SIVmac239^40^ were mixed with 13 μL 2 M CaCl2 and the total volume was filled up to 100 μL with water. This solution was added dropwise to 100 μL of 2× HBS. The transfection cocktail was vortexed for 5 s and added dropwise to the cells. The transfected cells were incubated for 8–16 h before the medium was replaced with fresh supplemented DMEM. 48 h post transfection, virus stocks were prepared by collecting the supernatant and centrifuging it at 1300 rpm for 3 min.

### TZM-bl Infection Assays

To determine infectious virus yield, 10,000 TZM-bl reporter cells/well were seeded in 96-well plates and infected with cell culture supernatants, under reduced 1% FCS conditions, in triplicates on the following day. Three days p.i., cells were lysed and β-galactosidase reporter gene expression was determined using the GalScreen Kit (Applied Bioscience) according to the manufacturer’s instructions with an Orion microplate luminometer (Berthold).

### Peptides

All peptides were commercially obtained from KE Biochem (China) with a purity of ≥95%. The peptides were dissolved in dimethyl sulfoxide (DMSO, Sigma-Aldrich, Hamburg, Germany) at a stock concentration of 50 mM and further diluted in phosphate-buffered saline (PBS) or Dulbecco’s Modified Eagle Medium (DMEM) cell culture media before use.

### Infection Inhibition

10,000 TZM-bl reporter cells were seeded in 96-well plates in 100 μL of supplemented DMEM. The following day, the medium was changed to reduced 1% FCS-supplemented DMEM, and serially diluted peptide was added to the cells. The cells were subsequently infected with 10 μL of infectivity-normalized virus stocks. At 3 days postinfection, the supernatant was removed, and β-galactosidase activity was measured as previously described.

### ReaxFF Based Molecular Dynamics Simulations

The molecular dynamics simulations of this study use the ReaxFF approach,^41^ which includes bond order-dependent energy terms that dynamically adapt to the local atomic environment. The C/H/O/N glycine/water parameters developed by Rahaman et al. and extended by Monti et al. were used.^42,43^ Rigorous tests confirmed the accuracy and transferability of the force field with a training set based on DFT-B3LYP/6-311++G** calculations of amino acid structures.^42,44^ The simulations were also compared against the classical ff99SB force field from the AMBER family.^45^ Based on the structure of the complex between the HIV-1 gp41 FP and VIR-165 from the Protein Data Bank:^46^ 2JNR (https://www.rcsb.org/structure/2JNR), the initial atomic positions were obtained. Equilibration (300 K for 0.5 ns) was performed by ReaxFF simulations within the Amsterdam Modeling Suite 2020 (http://www.scm.com). Based on the equilibrated structure, the amino acids from VIRIP were replaced by corresponding amino acids. After additional equilibration (300 K for 0.5 ns), ReaxFF simulations were performed within the NVT ensemble over 25 ps while the system was coupled to a Berendsen heat bath (*T* = 300 K with a coupling constant of 100 fs). The interaction energy was obtained by averaging over these simulations. UCSF Chimera^47^ was used for all visualizations.

### Hemolysis Assay

Fresh human blood, collected by venipuncture, was centrifuged (10 min, 1000×*g*, 4 °C) to pellet erythrocytes, which were then washed three times and resuspended (1:10) in DPBS. Peptides were serially diluted in a 96-well plate in DPBS. To 90 μL of peptide dilution, 10 μL of erythrocyte suspension was added. Following a 1-h incubation at 37 °C, while shaking at 500 rpm, the plates were centrifuged (5 min, 1000×*g*, 4 °C). Supernatant was transferred to transparent 96-well plates for absorbance measurement at λ = 405 nm, with full erythrocyte lysis indicated by the Triton-X control.

### MSA

Alignments of viral FP amino acid sequences were performed in Clustal Omega (https://www.ebi.ac.uk/Tools/msa/clustalo/) using the ClustalW 63 algorithm and an ordered input. The resulting phylogenetic tree was transferred to ITOL (https://itol.embl.de/) and visualized as a rectangular phylogenetic tree in default settings.

### Mass Spectrometry

Single or equimolarly mixed amounts of peptide, diluted in serum-reduced DMEM, were spotted on a 384-well plate and analyzed with an Axima Confidence MALDI-TOF mass spectrometer (Shimadzu, Japan) as follows: plate wells were coated with one microliter of 10 mg/mL CHCA (matrix) in HFBA/acetonitrile/2-propanol/water (v/v: 2.5/25/25/47.5), and the solvent evaporated; then, the sample (0.5 μL) was mixed with matrix (0.5 μL) onto the dry precoated well, and the solvent evaporated. The measurement was done in linear mode. An accelerating voltage of 20 kV was applied to the ion source, and the laser shots were automatically done following a regular circular raster of a diameter of 2000 μm and spacing of 200 μm on every well; 100 profiles were acquired per sample, and 20 shots were accumulated per profile. The measurement and MS data processing were performed with MALDI-MS Application Shimadzu Biotech Launchpad 2.9.8.1 software (Shimadzu, Japan).

### Zebrafish

For in vivo studies, wild-type zebrafish embryos (Danio rerio) were dechorionated at 24 h post fertilization (hpf) using digestion with 1 mg/mL Pronase (Sigma) in E3 medium (83 μM NaCl, 2.8 μM KCl, 5.5 μM 202 CaCl2, 5.5 μM MgSO4). In a 96-well plate, 3 embryos per well were exposed for 24 h to 100 μL of E3 containing VIRIP peptides at the concentrations indicated in the figures. Two independent assays were performed, each with 10 × 3 embryos. The peptide solvent (DMSO), diluted in E3, was used as negative control at the same amount as introduced by the peptide stock. As positive control for acute toxicity/cytotoxicity the pleurocidin antimicrobial peptide NRC-03 (GRRKRKWLRRIGKGVKIIGG AALDHL-NH2) was used at a concentration of 3 μM as described.^48^ Abamectin at a concentration of 3.125 μM was used as positive control for neurotoxicity.^49^ At 48 hpf (after 24 h of incubation) embryos were scored in a stereomicroscope for signs of cytotoxicity (lysis and/or necrosis), developmental toxicity (delay and/or malformations) or cardiotoxicity (heart edema and/or reduced or absent circulation). Each embryo was also touched with a needle and reduced or absent touch response (escape movements) was evaluated as signs of neurotoxicity if and only if no signs of acute toxicity were present in the same embryo. Embryos were categorized within each of these toxicity categories into several classes of severity.^35^ Chi-Square test was used to calculate whether the distribution of embryos into toxicity classes differed significantly between the PBS negative control and the test substances.

## Abbreviations

ACE2: angiotensin-converting enzyme 2
AUC: area under the curve
CD4: cluster of differentiation 4
CXCR4: C-X-C chemokine receptor type 4
DMEM: Dulbecco’s modified Eagle medium
DMSO: dimethyl sulfoxide
DNA: DNA
eV: electronvolt
FP: fusion peptide
FCS: fetal calf serum
GP41: glycoprotein 41
hpf: hours post fertilization
HIV: human immunodeficiency virus
HIV-1: human immunodeficiency virus type 1
HIV-2: human immunodeficiency virus type 2
IC50: half maximal inhibitory concentration
kDa: kilodalton
MS: mass spectrometry
NMR: nuclear magnetic resonance
PBS: phosphate-buffered saline
ps: picosecond
ReaxFF: reactive force field
SARS-CoV-2: severe acute respiratory syndrome coronavirus 2
SAR: structure–activity relationship
SIVmac: simian immunodeficiency virus infecting macaques
soVIRIP: size-optimized Virus Inhibitory Peptide
T20: Enfuvirtide (Fuzeon, HIV-1 entry inhibitor)
TF: transmitted-founder (virus strain)
TZM-bl: reporter cell line containing β-galactosidase under the HIV-1 LTR control
UCSF: University of California, San Francisco
VIRIP: Virus Inhibitory Peptide

## References

[ref1] Khalily M. P.; Soydan M. Peptide-Based Diagnostic and Therapeutic Agents: Where We Are and Where We Are Heading?. Chem. Biol. Drug Des 2023, 101 (3), 772–793. 10.1111/cbdd.14180. 36366980

[ref2] Wang L.; Wang N.; Zhang W.; Cheng X.; Yan Z.; Shao G.; Wang X.; Wang R.; Fu C. Therapeutic Peptides: Current Applications and Future Directions. Sig Transduct Target Ther 2022, 7 (1), 1–27. 10.1038/s41392-022-00904-4. PMC8844085 35165272

[ref3] Muttenthaler M.; King G. F.; Adams D. J.; Alewood P. F. Trends in Peptide Drug Discovery. Nat. Rev. Drug Discov 2021, 20 (4), 309–325. 10.1038/s41573-020-00135-8. 33536635

[ref4] Iglesias V.; Bárcenas O.; Pintado-Grima C.; Burdukiewicz M.; Ventura S. Structural Information in Therapeutic Peptides: Emerging Applications in Biomedicine. FEBS Open Bio 2025, 15 (2), 254–268. 10.1002/2211-5463.13847. PMC11788753 38877295

[ref5] Barman P.; Joshi S.; Sharma S.; Preet S.; Sharma S.; Saini A. Strategic Approaches to Improvise Peptide Drugs as Next Generation Therapeutics. Int. J. Pept Res. Ther 2023, 29 (4), 61 10.1007/s10989-023-10524-3. 37251528 PMC10206374

[ref6] Luo X.; Chen H.; Song Y.; Qin Z.; Xu L.; He N.; Tan Y.; Dessie W. Advancements, Challenges and Future Perspectives on Peptide-Based Drugs: Focus on Antimicrobial Peptides. European Journal of Pharmaceutical Sciences 2023, 181, 106363 10.1016/j.ejps.2022.106363. 36529161

[ref7] Rossino G.; Marchese E.; Galli G.; Verde F.; Finizio M.; Serra M.; Linciano P.; Collina S. Peptides as Therapeutic Agents: Challenges and Opportunities in the Green Transition Era. Molecules 2023, 28 (20), 7165 10.3390/molecules28207165. 37894644 PMC10609221

[ref8] Tsomaia N. Peptide Therapeutics: Targeting the Undruggable Space. Eur. J. Med. Chem. 2015, 94, 459–470. 10.1016/j.ejmech.2015.01.014. 25591543

[ref9] Schütz D.; Ruiz-Blanco Y. B.; Münch J.; Kirchhoff F.; Sanchez-Garcia E.; Müller J. A. Peptide and Peptide-Based Inhibitors of SARS-CoV-2 Entry. Adv. Drug Deliv Rev. 2020, 167, 47–65. 10.1016/j.addr.2020.11.007. 33189768 PMC7665879

[ref10] Freitas E. D.; Bataglioli R. A.; Oshodi J.; Beppu M. M. Antimicrobial Peptides and Their Potential Application in Antiviral Coating Agents. Colloids Surf., B 2022, 217, 112693 10.1016/j.colsurfb.2022.112693. PMC9262651 35853393

[ref11] Lee Y.-C. J.; Shirkey J. D.; Park J.; Bisht K.; Cowan A. J. An Overview of Antiviral Peptides and Rational Biodesign Considerations. BioDesign Research 2022, 2022, 9898241 10.34133/2022/9898241. 37850133 PMC10521750

[ref12] Todorovski T.; Kalafatovic D.; Andreu D. Antiviral Peptide-Based Conjugates: State of the Art and Future Perspectives. Pharmaceutics 2023, 15 (2), 357 10.3390/pharmaceutics15020357. 36839679 PMC9958607

[ref13] Al-Azzam S.; Ding Y.; Liu J.; Pandya P.; Ting J. P.; Afshar S. Peptides to Combat Viral Infectious Diseases. Peptides 2020, 134, 170402 10.1016/j.peptides.2020.170402. 32889022 PMC7462603

[ref14] Teissier E.; Penin F.; Pécheur E.-I. Targeting Cell Entry of Enveloped Viruses as an Antiviral Strategy. Molecules 2011, 16 (1), 221–250. 10.3390/molecules16010221. PMC6259279 21193846

[ref15] Mazzon M.; Marsh M. Targeting Viral Entry as a Strategy for Broad-Spectrum Antivirals. F1000Res. 2019, 8, 1628 10.12688/f1000research.19694.1. PMC6743247 31559009

[ref16] Majdoul S.; Compton A. A. Lessons in Self-Defence: Inhibition of Virus Entry by Intrinsic Immunity. Nat. Rev. Immunol 2022, 22 (6), 339–352. 10.1038/s41577-021-00626-8. 34646033 PMC8511856

[ref17] Dimitrov D. S. Virus Entry: Molecular Mechanisms and Biomedical Applications. Nat. Rev. Microbiol 2004, 2 (2), 109–122. 10.1038/nrmicro817. 15043007 PMC7097642

[ref18] White J. M.; Delos S. E.; Brecher M.; Schornberg K. Structures and Mechanisms of Viral Membrane Fusion Proteins. Crit Rev. Biochem Mol. Biol. 2008, 43 (3), 189–219. 10.1080/10409230802058320. 18568847 PMC2649671

[ref19] Lozada C.; Barlow T. M. A.; Gonzalez S.; Lubin-Germain N.; Ballet S. Identification and Characteristics of Fusion Peptides Derived From Enveloped Viruses. Front Chem. 2021, 9, 689006 10.3389/fchem.2021.689006. 34497798 PMC8419435

[ref20] Harrison S. C. Viral Membrane Fusion. Virology 2015, 479–480, 498–507. 10.1016/j.virol.2015.03.043. PMC4424100 25866377

[ref21] Wild C. T.; Shugars D. C.; Greenwell T. K.; McDanal C. B.; Matthews T. J. Peptides Corresponding to a Predictive Alpha-Helical Domain of Human Immunodeficiency Virus Type 1 Gp41 Are Potent Inhibitors of Virus Infection. Proc. Natl. Acad. Sci. U. S. A. 1994, 91 (21), 9770–9774. 10.1073/pnas.91.21.9770. 7937889 PMC44898

[ref22] Jiang X.; Jia Q.; Lu L.; Yu F.; Zheng J.; Shi W.; Cai L.; Jiang S.; Liu K. A Novel Bispecific Peptide HIV-1 Fusion Inhibitor Targeting the N-Terminal Heptad Repeat and Fusion Peptide Domains in Gp41. Amino Acids 2016, 48 (12), 2867–2873. 10.1007/s00726-016-2325-x. 27631437

[ref23] Xia S.; Lan Q.; Zhu Y.; Wang C.; Xu W.; Li Y.; Wang L.; Jiao F.; Zhou J.; Hua C.; et al. Structural and Functional Basis for Pan-CoV Fusion Inhibitors against SARS-CoV-2 and Its Variants with Preclinical Evaluation. Sig Transduct Target Ther 2021, 6 (1), 1–10. 10.1038/s41392-021-00712-2. PMC8320318 34326308

[ref24] Xia S.; Liu M.; Wang C.; Xu W.; Lan Q.; Feng S.; Qi F.; Bao L.; Du L.; Liu S.; et al. Inhibition of SARS-CoV-2 (Previously 2019-nCoV) Infection by a Highly Potent Pan-Coronavirus Fusion Inhibitor Targeting Its Spike Protein That Harbors a High Capacity to Mediate Membrane Fusion. Cell Res. 2020, 30 (4), 343–355. 10.1038/s41422-020-0305-x. 32231345 PMC7104723

[ref25] Su X.; Huang Z.; Xu W.; Wang Q.; Xing L.; Lu L.; Jiang S.; Xia S. IgG Fc-Binding Peptide-Conjugated Pan-CoV Fusion Inhibitor Exhibits Extended In Vivo Half-Life and Synergistic Antiviral Effect When Combined with Neutralizing Antibodies. Biomolecules 2023, 13 (9), 1283 10.3390/biom13091283. 37759683 PMC10526447

[ref26] Münch J.; Ständker L.; Adermann K.; Schulz A.; Schindler M.; Chinnadurai R.; Pöhlmann S.; Chaipan C.; Biet T.; Peters T.; et al. Discovery and Optimization of a Natural HIV-1 Entry Inhibitor Targeting the Gp41 Fusion Peptide. Cell 2007, 129 (2), 263–275. 10.1016/j.cell.2007.02.042. 17448989

[ref27] Blumenthal R.; Dimitrov D. S. Targeting the Sticky Fingers of HIV-1. Cell 2007, 129 (2), 243–245. 10.1016/j.cell.2007.04.005. 17448985

[ref28] van Gils M. J.; Sanders R. W. Hitting HIV’s Harpoon. Immunity 2018, 49 (1), 14–15. 10.1016/j.immuni.2018.07.003. 30021141

[ref29] Venken T.; Krnavek D.; Münch J.; Kirchhoff F.; Henklein P.; De Maeyer M.; Voet A. An Optimized MM/PBSA Virtual Screening Approach Applied to an HIV-1 Gp41 Fusion Peptide Inhibitor. Proteins 2011, 79 (11), 3221–3235. 10.1002/prot.23158. 21989940

[ref30] Schauenburg D.; Zech F.; Heck A. J.; von Maltitz P.; Harms M.; Führer S.; Alleva N.; Münch J.; Kuan S. L.; Kirchhoff F.; et al. Peptide Bispecifics Inhibiting HIV-1 Infection by an Orthogonal Chemical and Supramolecular Strategy. Bioconjug Chem. 2023, 34 (9), 1645–1652. 10.1021/acs.bioconjchem.3c00314. 37665137 PMC10515486

[ref31] Gonzalez E.; Ballana E.; Clotet B.; Esté J. A. Development of Resistance to VIR-353 with Cross-Resistance to the Natural HIV-1 Entry Virus Inhibitory Peptide (VIRIP). Aids 2011, 25 (13), 1575–1583. 10.1097/QAD.0b013e328348a733. 21572303

[ref32] Müller J. A.; Glöckle A.; Gawanbacht A.; Geyer M.; Münch J.; Kirchhoff F. Reduced Susceptibility to VIRIP-Based HIV-1 Entry Inhibitors Has a High Genetic Barrier and Severe Fitness Costs. Journal of Virology 2018, 92 (17), 733–751. 10.1128/JVI.00733-18. PMC6096827 29925662

[ref33] Forssmann W. G.; The Y. H.; Stoll M.; Adermann K.; Albrecht U.; Barlos K.; Busmann A.; Canales-Mayordomo A.; Giménez-Gallego G.; Hirsch J.; et al. Short-Term Monotherapy in HIV-Infected Patients with a Virus Entry Inhibitor against the Gp41 Fusion Peptide. Science Translational Medicine 2010, 2 (63), 63re3 10.1126/scitranslmed.3001697. 21178138

[ref34] Wei X.; Decker J. M.; Liu H.; Zhang Z.; Arani R. B.; Kilby J. M.; Saag M. S.; Wu X.; Shaw G. M.; Kappes J. C. Emergence of Resistant Human Immunodeficiency Virus Type 1 in Patients Receiving Fusion Inhibitor (T-20) Monotherapy. Antimicrob. Agents Chemother. 2002, 46 (6), 1896–1905. 10.1128/AAC.46.6.1896-1905.2002. 12019106 PMC127242

[ref35] Harms M.; Hansson R. F.; Gilg A.; Almeida-Hernández Y.; Löffler J.; Rodríguez-Alfonso A.; Habib M. M. W.; Albers D.; Ahmed N. S.; Abadi A. H.; Winter G.; Rasche V.; Beer A. J.; Weidinger G.; Preising N.; Ständker L.; Wiese S.; Sanchez-Garcia E.; Zelikin A. N.; Münch J. Development of N-Terminally Modified Variants of the CXCR4-Antagonistic Peptide EPI-X4 for Enhanced Plasma Stability. J. Med. Chem. 2023, 66 (22), 15189–15204. 10.1021/acs.jmedchem.3c01128. 37940118 PMC10682998

[ref36] Haddadzadegan S.; Dorkoosh F.; Bernkop-Schnürch A. Oral Delivery of Therapeutic Peptides and Proteins: Technology Landscape of Lipid-Based Nanocarriers. Adv. Drug Delivery Rev. 2022, 182, 114097 10.1016/j.addr.2021.114097. 34999121

[ref37] Adachi A.; Gendelman H. E.; Koenig S.; Folks T.; Willey R.; Rabson A.; Martin M. A. Production of Acquired Immunodeficiency Syndrome-Associated Retrovirus in Human and Nonhuman Cells Transfected with an Infectious Molecular Clone. Journal of Virology 1986, 59 (2), 284–291. 10.1128/jvi.59.2.284-291.1986. 3016298 PMC253077

[ref38] Ochsenbauer C.; Edmonds T. G.; Ding H.; Keele B. F.; Decker J.; Salazar M. G.; Salazar-Gonzalez J. F.; Shattock R.; Haynes B. F.; Shaw G. M.; et al. Generation of Transmitted/Founder HIV-1 Infectious Molecular Clones and Characterization of Their Replication Capacity in CD4 T Lymphocytes and Monocyte-Derived Macrophages. Journal of Virology 2012, 86 (5), 2715–2728. 10.1128/JVI.06157-11. 22190722 PMC3302286

[ref39] Clavel F.; Guyader M.; Guétard D.; Sallé M.; Montagnier L.; Alizon M. Molecular Cloning and Polymorphism of the Human Immune Deficiency Virus Type 2. Nature 1986, 324 (6098), 691–695. 10.1038/324691a0. 3025743

[ref40] Regier D. A.; Desrosiers R. C. The Complete Nucleotide Sequence of a Pathogenic Molecular Clone of Simian Immunodeficiency Virus. AIDS Research and Human Retroviruses 1990, 6 (11), 1221–1231. 10.1089/aid.1990.6.1221. 2078405

[ref41] van Duin A. C. T.; Dasgupta S.; Lorant F.; Goddard W. A. III ReaxFF: A Reactive Force Field for Hydrocarbons. J. Phys. Chem. A 2001, 105 (41), 9396–9409. 10.1021/JP004368U.

[ref42] Monti S.; Corozzi A.; Fristrup P.; Joshi K. L.; Shin Y. K.; Oelschlaeger P.; Van Duin A. C. T.; Barone V. Exploring the Conformational and Reactive Dynamics of Biomolecules in Solution Using an Extended Version of the Glycine Reactive Force Field. Phys. Chem. Chem. Phys. 2013, 15 (36), 15062 10.1039/c3cp51931g. 23925839

[ref43] Rahaman O.; Van Duin A. C. T.; Goddard W. A.; Doren D. J. Development of a ReaxFF Reactive Force Field for Glycine and Application to Solvent Effect and Tautomerization. J. Phys. Chem. B 2011, 115 (2), 249–261. 10.1021/jp108642r. 21166434 PMC3042430

[ref44] Lee C.; Yang W.; Parr R. G. Development of the Colle-Salvetti Correlation-Energy Formula into a Functional of the Electron Density. Phys. Rev. B 1988, 37 (2), 785–789. 10.1103/PhysRevB.37.785. 9944570

[ref45] Hornak V.; Abel R.; Okur A.; Strockbine B.; Roitberg A.; Simmerling C. Comparison of Multiple Amber Force Fields and Development of Improved Protein Backbone Parameters. Proteins 2006, 65 (3), 712–725. 10.1002/prot.21123. 16981200 PMC4805110

[ref46] Berman H. M.; Westbrook J.; Feng Z.; Gilliland G.; Bhat T. N.; Weissig H.; Shindyalov I. N.; Bourne P. E. The Protein Data Bank. Nucleic Acids Res. 2000, 28 (1), 235–242. 10.1093/nar/28.1.235. 10592235 PMC102472

[ref47] Pettersen E. F.; Goddard T. D.; Huang C. C.; Couch G. S.; Greenblatt D. M.; Meng E. C.; Ferrin T. E. UCSF Chimera--a Visualization System for Exploratory Research and Analysis. J. Comput. Chem. 2004, 25 (13), 1605–1612. 10.1002/jcc.20084. 15264254

[ref48] Morash M. G.; Douglas S. E.; Robotham A.; Ridley C. M.; Gallant J. W.; Soanes K. H. The Zebrafish Embryo as a Tool for Screening and Characterizing Pleurocidin Host-Defense Peptides as Anti-Cancer Agents. Dis Model Mech 2011, 4 (5), 622–633. 10.1242/dmm.007310. 21729875 PMC3177944

[ref49] Raftery T. D.; Isales G. M.; Yozzo K. L.; Volz D. C. High-Content Screening Assay for Identification of Chemicals Impacting Spontaneous Activity in Zebrafish Embryos. Environ. Sci. Technol. 2014, 48 (1), 804–810. 10.1021/es404322p. 24328182

